# Evaluation and Proteomic Analysis of Lead Adsorption by Lactic Acid Bacteria

**DOI:** 10.3390/ijms20225540

**Published:** 2019-11-06

**Authors:** Shaoli Liu, Yi Zheng, Yimiao Ma, Abid Sarwar, Xiao Zhao, Tianqi Luo, Zhennai Yang

**Affiliations:** 1 Beijing Advanced Innovation Center for Food Nutrition and Human Health, Beijing Technology and Business University, Beijing 100048, China; shaolil@126.com (S.L.);; 2 Beijing Engineering and Technology Research Center of Food Additives, Beijing Technology and Business University, Beijing 100048, China

**Keywords:** lactic acid bacteria, *Lactobacillus plantarum*, lead, absorption, proteomics

## Abstract

Heavy metals are a growing threat to human health due to the resulting damage to the ecology; the removal of heavy metals by lactic acid bacteria (LAB) has been a focus of many studies. In this study, 10 LAB strains were evaluated for their ability to absorb and tolerate lead. *Lactobacillus plantarum* YW11 was found to possess the strongest ability of lead absorbing and tolerance, with the rate of absorption as high as 99.9% and the minimum inhibitory concentration of lead on YW11 higher than 1000 mg/L. Based on the isobaric tags for relative and absolute quantitation (iTRAQ) proteomics analysis of YW11, a total of 2009 proteins were identified both in the lead-treated strain and the control without the lead treatment. Among these proteins, 44 different proteins were identified. The abundance of 25 proteins increased significantly, and 19 proteins decreased significantly in the treatment group. These significantly differential abundant proteins are involved in the biological processes of amino acid and lipid metabolism, energy metabolism, cell wall biosynthesis, and substance transport. This study contributed further understanding of the molecular mechanism of *L. plantarum* in the binding and removal of lead to explore its potential application in counteracting heavy metal pollution of environment, food, and other fields.

## 1. Introduction

Along with rapid industrial development and urban modernization, heavy metal pollution has become a major environmental problem worldwide [1,2]. Heavy metals are difficult to be degraded or transformed in the natural environment, resulting in their persistent accumulation in crops, aquatic products, fruits, and vegetables. Biological enrichment of these heavy metals through the food chain may ultimately cause serious harm to human health [3]. Among the heavy metals, lead, mercury, and cadmium are commonly present in contaminated wastewater, and lead is known for its high environmental impact and toxicity [4,5]. It has been reported that very low concentrations of lead may cause toxic effects on the immune system, nervous system, hematopoietic system, liver, kidney, brain, and other organs, leading to diseases such as anorexia, chronic kidney disease, neuronal injury, and Alzheimer’s disease [6,7,8,9]. Studies have shown that lead can affect a variety of cellular processes, such as cell signaling, cell adhesion, protein folding and maturation, apoptosis, ionic transportation, enzyme regulation, etc. [10]. Therefore, effective removal of heavy metals is vital in maintaining ecological balance and human health.

Heavy metal contamination can be removed by different methods including physical deposition, chemical treatment, and biological adsorption [11,12,13,14]. Removal of heavy metals by microbial adsorption has been considered with advantages of high efficiency, nontoxicity, environmental protection, and economy without secondary pollution, and this has drawn increased research attention [15]. During the last decade, many studies have been carried out on the use of lactic acid bacteria (LAB), possessing generally recognized as safe (GRAS) status, to absorb a variety of heavy metals [16,17,18,19]. LABs are widely used in food processing, microecological preparation, pharmaceutical development, and other fields [20,21,22]. LABs are the intestinal microorganisms in human and animal intestines, and many of the strains have been characterized as probiotics with beneficial health functions such as alleviation of lactose intolerance, improvement of immunity, and effective prevention/treatment of diseases [23]. Some LAB strains have been shown to effectively absorb and tolerate heavy metals, thus repairing the oxidative stress injury of the intestine, improving the intestinal function, and preventing absorption of heavy metals of the host [24,25].

Currently, *Lactobacillus*, *Bifidobacterium*, *Enterococcus*, and *Propionibacterium* have been found to possess the ability to absorb lead [16,17,18,26]. *Bifidobacterium longum* 46 had a maximum absorption amount of 175.7 mg/g of dry matter for lead in phosphate buffer solution (PBS) [26]. Follow-up studies showed that *B. longum* 46 and *Lactobacillus fermentum* ME3 were able to absorb a large number of lead particles through the electrostatic action on the cell surface [26]. The lead adsorption rates of *Enterococcus faecium* M74 and *E. faecium* EF031 were 42.9–93.1% and 66.9–98.9%, respectively [18]. *Lactobacillus reuteri* Pb71-1 isolated from the heavy metal-contaminated silt samples had a 59% removal rate for lead ions in de Man, Rogosa, and Sharpe (MRS) [19]. *E. faecium* Pb12 isolated from sludge in coastal aquaculture fields effectively absorb lead at 0.0460 mg/h/g of cells (wet weight) in the fish intestinal tract through the surface adsorption of cells [27]. *Lactobacillus plantarum* CCFM8661 had the highest lead binding ability, and the biosorption level reached 49.551 mg/g of dry biomass [25]. *L. plantarum* 70810 isolated from Chinese paocai was found to produce large amounts of exopolysaccharide that could adsorb 276.44 mg/g of lead [28].

To date, most of the studies on heavy metal absorption by LAB focus on screening of strains and comparison of absorption characteristics, but further elaboration of the absorption mechanism by LAB has not been well studied. Thus, the aims of this study were not only to evaluate the absorption and tolerance of lead by LAB but also to analyze the proteins that are involved in regulation of the absorption process by proteomics method. The present study provides a more systematic and in-depth understanding of the molecular mechanism involved in absorption of lead by LAB.

## 2. Results

### 2.1. Lead Absorption Capacity and Tolerance of LAB

**Figure 1:**
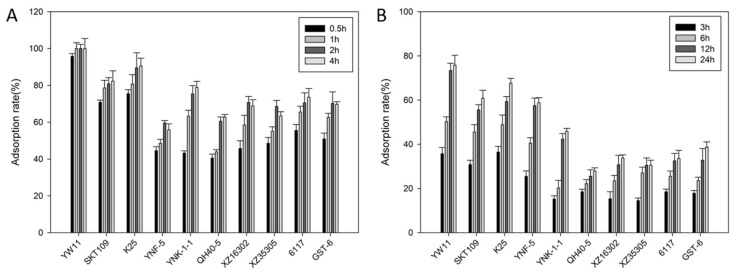
Lead absorption rates from aqueous solution (**A**) and MRS (de Man, Rogosa, and Sharpe) broth (**B**) by different lactic acid bacteria strains: Bars show the mean ± SE (standard error) of three biological replicates.

Ten LAB strains previously isolated from traditional fermented dairy products were compared for ability of lead absorption in the aqueous solution and MRS broth (Figure 1A,B). The results showed that the total metal removal pattern of the selected LAB strains in the MRS broth was similar to that in the aqueous solution but that the adsorption capacity of each strain in the MRS medium was much lower than that in the aqueous solution. Furthermore, the absorption capacity of *L. plantarum* strains (70–100% in the aqueous solution and 60–78% in MRS broth) was generally stronger than that of *Lactococcus lactis*, *Lactobacillus casei*, and *Streptococcus thermophilus* strains (60–80% in the aqueous solution and 30–50% in MRS broth). In addition, it was also observed that, in aqueous solution, the adsorption rate decreased slightly after incubation for 4 h for the *L. lactis* strains while, in MRS medium, the adsorption capacity of all the strains increased continuously during the whole period of incubation. Remarkably, the adsorption rates of *L. plantarum* YW11 reached 95.76% at 0.5 h in water and 78% in MRS broth. Meanwhile, the lead ions in the aqueous solution could not be detected by inductively coupled plasma-mass spectrometry (ICP-MS) at 1 h. Obviously, the lead adsorption capacity of YW11 was significantly higher than that of the other LAB strains tested.

Tolerance to lead by the 10 LAB strains was further assayed as shown in Table 1. Two *L. lactis* strains (YNK-1-1 and XZ16302), three *L. plantarum* strains (YW11, SKT109, and K25), *L. casei* 6117, and *S. thermophilus* GST-6 were resistant to lead ions at 100 mg/L. *L. plantarum* YW11 showed the highest resistance to lead, and the minimum inhibitory concentration (MIC) was higher than 1000 mg/L.

### 2.2. Effect of Lead on the Growth of LAB

**Figure 2:**
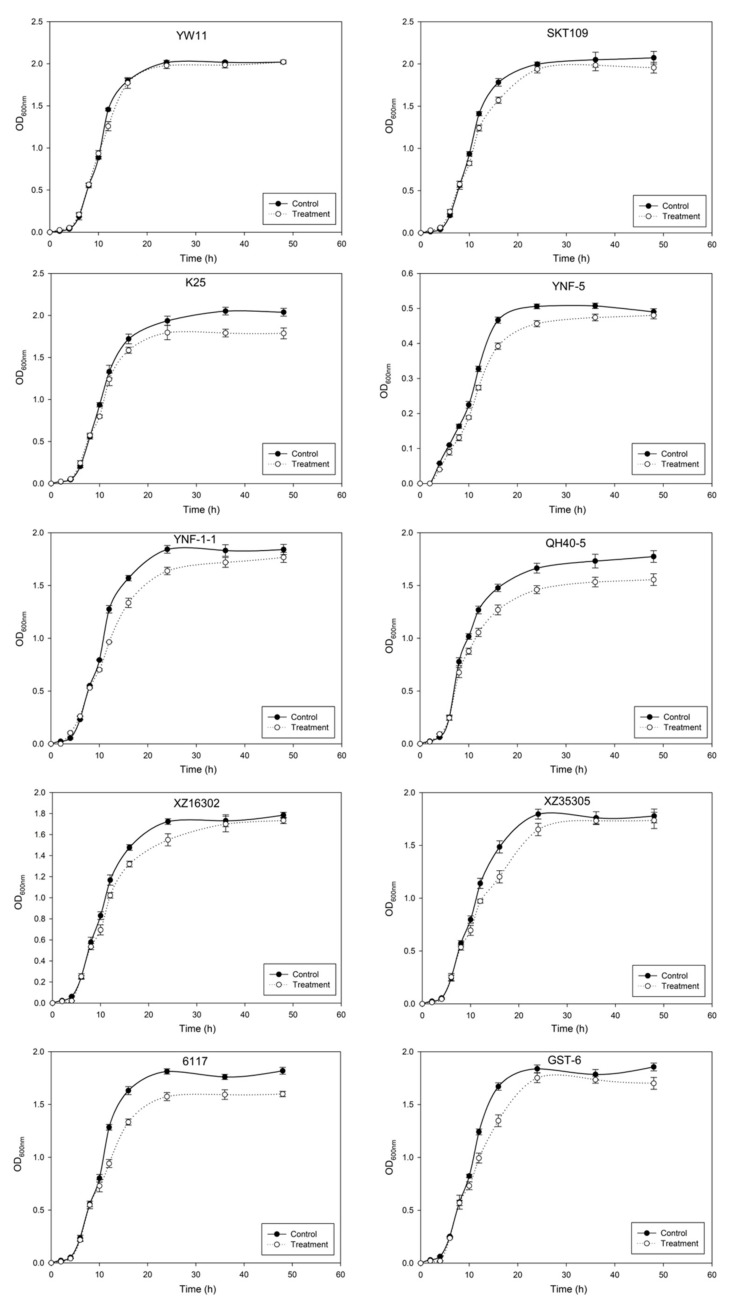
Effect of lead on the growth of different lactic acid bacterial strains. Control: lactic acid bacteria (LAB) strains in lead-free MRS medium; treatment: LAB strains in MRS medium containing lead ions (100 mg/L). OD_600nm_: absorbance of medium at optical density 600nm.

To verify the effect of lead on bacterial growth, the 10 LAB strains were inoculated in MRS liquid medium containing 50 mg/L lead ions and their growth curves were determined (Figure 2). Lead ions had different effects on the growth of LAB, especially in the later logarithmic phase (or the early stationary phase). Apparently, the growth of *L. plantarum* YW11 was least affected by lead ions.

On the basis of the results of lead binding and tolerance as described above, the most promising strain, *L. plantarum* YW11, which had the highest lead binding capacity and effective tolerance to lead inhibition, was selected for use in the follow-up experiments.

### 2.3. Scanning Electron Microscopy and Energy Spectrum Scanning

**Figure 3:**
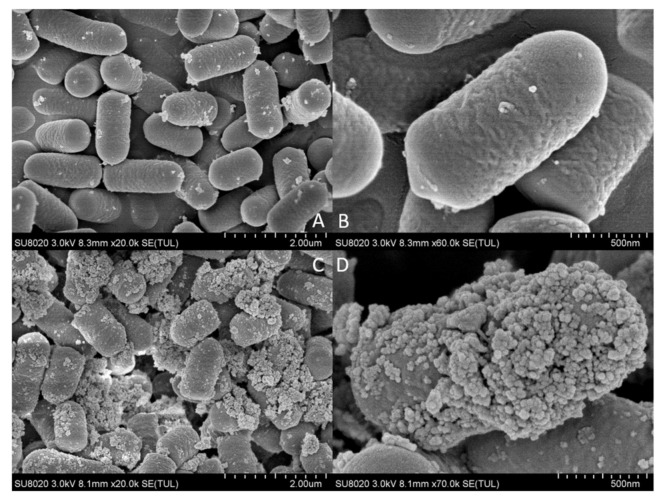
Lead-binding photomicrographs of *L. plantarum* YW11 by SEM. (**A**,**B**) Observation of YW11 in lead-free MRS medium; (**C**,**D**) observation of YW11 in MRS medium containing lead ions (100 mg/L).

**Figure 4:**
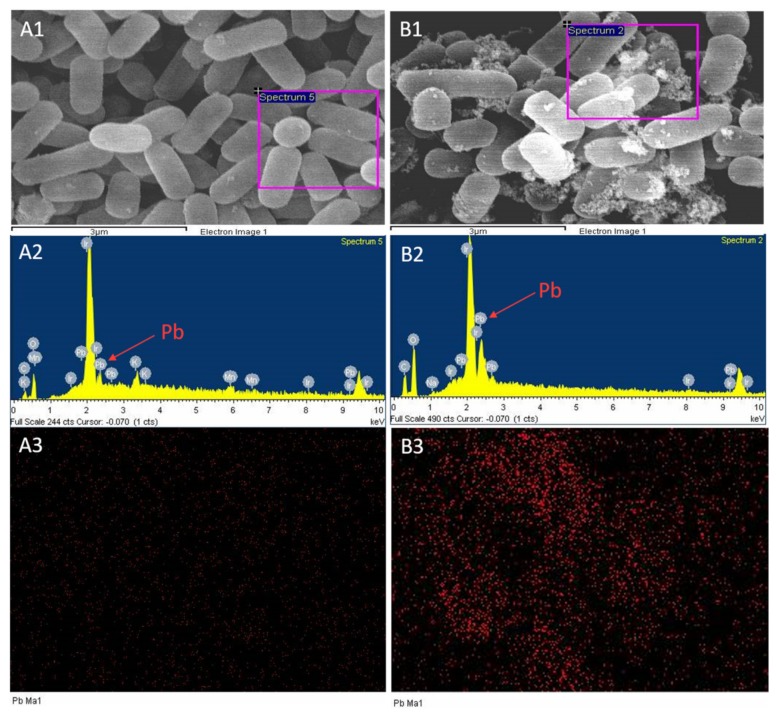
Energy dispersive spectroscopy (EDS) mappings of lead binding by *L. plantarum* YW11. (**A1–3**) Observation of YW11 in lead-free MRS medium; (**B1–3**) observation of YW11 in MRS medium containing lead ions (100 mg/L). (**1**) Photomicrographs of *L. plantarum* YW11 by SEM; (**2**) EDS spectrum of YW11; and (**3**) EDS mapping of lead corresponding to (**1**).

Adsorption of lead by *L. plantarum* YW11 was observed by SEM after incubation in MRS medium containing 100 mg/L of lead ions, using the cultured cells in MRS medium without lead ions as the control (Figure 3). The SEM images clearly showed that there was substantial accumulation on the surface of YW11 treated with lead ions ( Figure 3C,D and Figure 4B1) in comparison with that of the control (Figure 3A,B and Figure 4A1). According to the EDS analyses on the elements and structures of the particles, the core particles were composed of Ir, Pb, O, and C (Figure 4A2,B2). The weight percentaged of lead were 14.58% and 30.99% in the control group and the treatment group, respectively. However, there was little change in the weight percentage of other elements in both groups. Meanwhile, EDS mapping showed that the distribution of lead in the control group was almost invisible while that in the treatment group was higher (Figure 4A3,B3). These results confirmed that the accumulation on the bacterial surface was lead and that YW11 had strong adsorption capacity for lead.

### 2.4. Quality Control Evaluation and Statistics of Proteomics

In order to understand the molecular mechanism of *L. plantarum* YW11 adsorbing lead ions, a proteomics-based approach was used to identify differentially expressed proteins of the strain upon treatment with lead ions. After isobaric tags for relative and absolute quantitation (iTRAQ) labeling, the peptide information of each sample from the treatment group and the control was obtained by mass spectrometry.

All peptides were statistically analyzed. The error analysis of all peptides obtained by mass spectrometry showed that the error was within 5 parts per million (ppm), which proved the reliability of the identification results (Figure S1). The peptide number distribution diagram is shown in Figure S2. Most identified proteins contained less than 10 peptides. Both Figures S3 and S4 showed a good correlation between 1, 2, and 3 in the control group and between 4, 5, and 6 in the treatment group. These results indicated a high repeatability of the three samples from each group. In this experiment, a total of 329,847 spectra was detected by mass spectrometry and 38,335 spectra were identified. The identified peptide number was 14,300. After removing the redundancy, the protein group number was 2009. Statistical analysis showed that 99.7% of the protein was within 100 kDa and that 41.1% of the protein was within 21–41 kDa (Figure S5).

### 2.5. Statistics of Differentially Expressed Proteins

Protein expression by *L. plantarum* YW11 treated with lead ions was compared with that of the control treated without lead ions. There were 44 proteins significantly differentially expressed (*p* < 0.05, FC > 1.5 or < −1.5). Among them, 25 proteins were significantly upregulated and 19 proteins significantly downregulated in the treatment group.

### 2.6. Gene Ontology (GO) and Kyoto Encyclopedia of Genes and Genomes (KEGG) Analyses of Differentially Expressed Proteins

**Figure 5:**
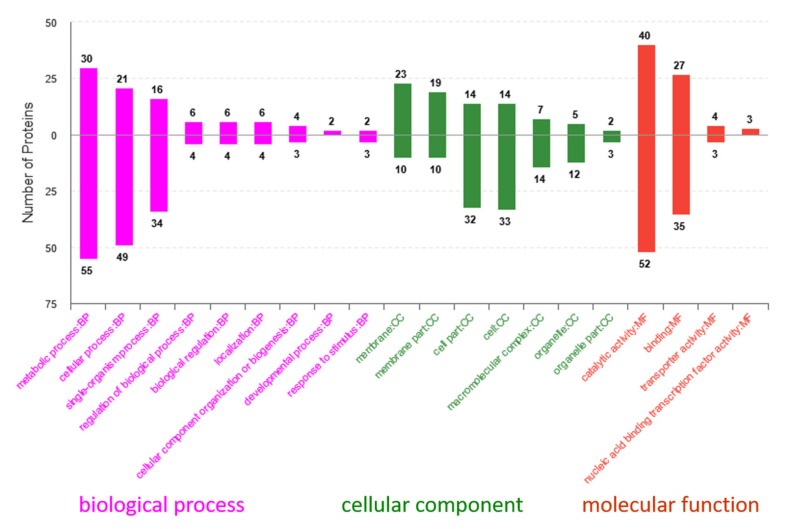
Gene ontology annotation of distribution analysis: The upward column represents the upregulated protein amount in the treatment group, and the downward column represents the downregulated protein amount in the treatment group. BB: biological process; CC: cellular component; MF: molecular function.

According to the annotation analysis of Uniprot database and gene ontology, the differential proteins played important roles in biological processes and different molecular functions and as cellular components (Figure 5). As shown in Figure 5, the proteins significantly upregulated in the GO secondary classification were mainly involved in metabolic processes, cellular processes, membrane transportation, catalytic activity, and binding.

GO enrichment results showed that the differential proteins were significantly enriched to 15 GO terms (*p* < 0.01) and that many of the proteins were involved in regulatory functions of lactobacilli in stress environments. These regulatory functions include teichoic acid d-alanylation, d-alanyl carrier activity, *C*-acetyltransferase activity, ACP phosphopantetheine attachment site binding, fatty acid biosynthesis, and phosphate ion binding.

**Figure 6:**
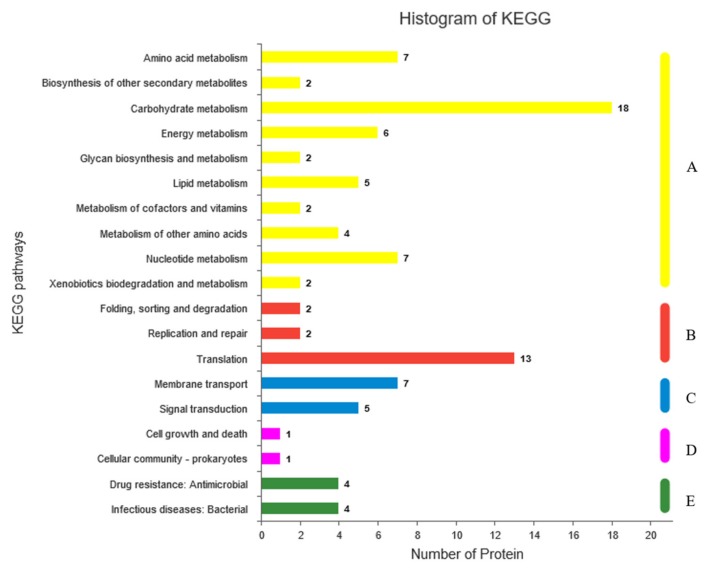
Kyoto Encyclopedia of Genes and Genomes (KEGG) annotation of distribution analysis of the 5 branches in the KEGG pathways including metabolism (**A**), genetic information processing (**B**), environmental information processing (**C**), cellular processes (**D**), and human diseases (**E**).

To further explore the metabolic pathways in which the differential proteins might be involved, KEGG functional annotation and enrichment analysis were performed (Figure 6). KEGG functional annotation showed that the differentially expressed proteins mainly concentrated in amino acid metabolism, carbohydrate metabolism, energy metabolism, membrane transport, signal transduction, lipid metabolism, etc. Enrichment analysis showed that the differential proteins were enriched to 20 metabolic pathways, including 5 significantly enriched metabolic pathways (*p*-value < 0.001). Among them, there were pathways associated with microbial stress: amino acid metabolism, fatty acid biosynthesis, lipopolysaccharide biosynthesis, two-component system, butanoate metabolism, and phosphotransferase system.

### 2.7. Differential Protein Expression Pattern Analysis

To identify proteins that might be involved in the lead-stress response, a total of 44 proteins was selected as significantly differentially expressed proteins (with FC > 1.5 or FC < −1.5 and *p* < 0.05) when comparing the treatment and control groups (Table 2). These proteins were categorized into global stress response, carbohydrate metabolism, translation, membrane and extracellular proteins, etc. based on the KEGG pathway analysis and their annotated functions in the Uniprot database. In this study, we found that 9 proteins involved in global stress response including 3 proteins, such as 2 flavin mononucleotide (FMN) binding protein and A0A385PQP5, upregulated and 6 proteins (A0A0R1UML2, A0A1E3KN19, D7V968, etc.) downregulated, indicating that lead can significantly inhibit the expression of proteins that resist the toxicity of lead. These upregulated proteins probably play a crucial role in regulating YW11 for normal growth. In addition, 3 proteins were involved in carbohydrate metabolism including 1 upregulated protein (A0A1S0RZ68) and 1 downregulated protein (T5JT98).

Compared with the control, higher abundances of proteins belonging to transporter (PstS), transcriptional regulation (nrdR), membrane proteins (peptidoglycan-binding protein, adhesin, cell wall anchor domain-containing protein, etc.), global stress response (FMN-binding protein), extracellular proteins (A0A0G9FAG5 and A0A2I0ZH16), carbohydrate metabolism (A0A1S0RZ68), and some uncharacterized proteins (A0A162GHW5, A0A0G9F7Y4, etc.) could be observed in the treatment group. On the other hand, all the proteins involved in translation (rpsU, rplW, rpmI, etc.) showed lower abundances in the treatment group. From these results, we could infer that all the transporter, the membrane, cell surface, and extracellular proteins with significantly upregulated expression was closely connected with the capacity of YW11 absorbing lead. Hence, the function of these proteins will be studied in subsequent research.

## 3. Discussion

Biosorption of lead by LAB might be associated with physical and chemical adsorption involving ion exchange processes on the bacterial surface [26,28,29]. Halttunen et al. [26] suggested that binding of lead occurs passively on the surface of bacteria rather than by accumulation inside the cell due to rapid removal of metals. Ibrahim and others [15] tested the binding efficiency of lead by *Lactobacillus rhamnosus* LC705, *Propionibacterium freudenreichii* subsp. *shermanii* JS, and a mixture of these strains, and they found that the bulk of the binding occurred within the first 5 min of incubation. In this study, the lead tolerant *L. plantarum* YW11 was found to efficiently bind lead within 2 hours of incubation and that the adsorption rate did not increase from 2 to 6 h in the sterile ultrapure water (Figure 1A). In MRS broth, *L. plantarum* YW11 also exhibited higher capability of lead adsorption than the other LAB strains tested with a similar trend of lead adsorption to that in water (Figure 1B), as previously reported [19]. It was speculated that the organic substances of MRS broth prevented LAB from absorbing lead. However, some *Lactobacillus* strains were reported to be incapable of removing Pb from MRS broth probably due to differences in bacterial surface structure and functional groups among the different strains [30]. 

As indicated in Figure 2, presence of lead ions influenced differently the growth and proliferation of the different LAB strains tested. These strains seemed to adapt themselves to maintain growth by developing resistance mechanisms under the stress of the heavy metal ions. Among the 10 LAB strains tested, *L. plantarum* YW11 demonstrated the highest tolerance to Pb (MIC > 1000 mg/L) as indicated by the MIC results (Table 1). At the same time, the growth of YW11 was also the least affected by lead. This is consistent with previous research [27,30]. Bhakta et al. showed that Pb-resistant Lactobacillus strains more likely demonstrated increased Pb removal efficiency [19]. Therefore, *L. plantarum* YW11 that acquired relatively strong Pb-resistant capacity could be a potential Pb remover.

Proteomics analysis showed that the significantly upregulated proteins in *L. plantarum* YW11 after lead exposure were mostly cell surface proteins such as S-layer and the membrane proteins, i.e., peptide-glycan binding proteins (A0A199QM58 and A0A2K7QYX4), lipoproteins (A0A0R1V4H5 and A0A0G9F7Y4), and other extracellular proteins (e.g., A0A0G9FAG5). Previously, the S-layer was reported to be essential for the adsorption of some *L. plantarum* phages [31,32]. Studies also revealed that two *L. kefir* strains, CIDCA 8348 and JCM 5818, after metal absorption were studied by electron microscopy and Fourier transform infrared spectroscopy (FTIR), showing precipitation of metals in the cell S-layer that caused changes in the secondary structure and protein arrangement of the S-layer [33]. The follow-up study disclosed that microorganisms without S-layer proteins were more prone to the detrimental effect of lead, suggesting that S-layer proteins acted as a protective rather than as a sequestrant layer [34]. In lactic acid bacteria, lead was shown to mainly affect the space structure of biofilm by affecting the S-layer proteins, thus inhibiting the formation of biofilm [35]. Therefore, the resistance of *L. plantarum* YW11 to lead stress of this study might involve increased expression of S-layer proteins to maintain cell structure and normal growth after lead exposure. In addition, some proteins located in the cell wall/plasma membrane, e.g., cytoskeletal protein (A0A165XVF0) that was 1.55-fold upregulated upon lead exposure (Table 2), played an important role in maintaining stability of the cell wall/plasma membrane and structural integrity of the cells [36]. Furthermore, the proteins involved in transport and carbohydrate metabolism were upregulated to varying degrees. The ATP-binding cassette (ABC) transporters (PstS and A0A151G577) were 1.7/1.54-fold upregulated, suggesting its active role in lead adsorption of YW11. ABC transporters, especially PstS, play important roles in transporting various substrates, such as ions, amino acids, peptides, sugars, and other hydrophilic molecules, across the cellular membrane [37,38,39]. ABC transporters of LAB were essential for cell viability under biotic and abiotic stresses [40,41,42], whereas the function of ABC proteins in the absorption of metal ions by lactobacilli as observed in this study was not reported earlier. Further study on their specific roles in lead absorption by LAB is needed. Our results also showed that glycosyl hydrolase (A0A1S0RZ68) mainly involved in exopolysaccharide biosynthesis and metabolism was 1.89-fold upregulated, indicating possibility of increased exopolysaccharide formation by *L. plantarum* YW11 under lead stress. Strain YW11 was previously reported to be capable of producing an exopolysaccharide [43]. Production of exopolysaccharide could be induced by environmental stresses [44]. Exopolysaccharides could trap and transport heavy metals rapidly into the cell membrane and release them gradually [19]. Feng et al. reported that the exopolysaccharide of *L. plantarum* 70810 had good adsorption properties for lead [28]. It seemed that efficient production of exopolysaccharide by *L. plantarum* YW11 contributed significantly to its remarkable property of lead adsorption.

Therefore, *L. plantarum* YW11 displayed a complex biological network to tackle the lead stress by a mechanism involving changes in metabolic pathways of amino acids and lipids, energy metabolic pattern, global stress response, and membrane transport, as described above. In these pathways, YW11 was likely to maintain the integrity of cell structure and metabolism by upregulating the expression of membrane proteins, extracellular proteins, proteins of carbohydrate metabolism, and global stress response, thus producing more substances conducive to adsorption of lead. There were reports that enzymes, glycoproteins, lipopolysaccharides, lipoproteins, and phospholipids were the active sites involved in metal-binding processes in microbes [45,46,47,48]. Some microorganisms that could accumulate heavy metals with ability to tolerate one or more metals exhibited enhanced transformational abilities, allowing the organism to lessen the toxic effect of the metal [49]. Apparently, the adsorption mechanism of lead by YW11 was related to a specific energy conservation, survival mode and repairing system, and enhanced protein synthesis ability. However, the exact mechanism of binding and detoxifying by *L. plantarum* YW11 under lead stress, e.g., key proteins, change of cell wall components, lead-binding sites, etc., needs to be further studied.

In conclusion, YW11 could cope with environmental stress with mild induction of the cellular defense and repair system, enabling the strain to survive lead exposure without drastic physiological response. In addition, YW11 had inherent superior lead-binding ability and effective cell wall structures, which promoted lead sequestration on the surface of the cell, preventing the uptake of this toxic metal into the cytoplasm. The results of this study provide significant insights into lead removal and mechanism of lead adsorption by LAB. However, this is a complex process and further work is needed to properly characterize the genes, proteins, and pathways involved.

## 4. Materials and Methods

### 4.1. Bacterial Strains and Culture Conditions

Ten LAB strains including 4 *L. plantarum* strains (YW11, SKT109, K25, and YNF-5) isolated from Tibet Kefir and 4 *Lactococcus lactis* strains (YNK-1-1, QH40-5, XZ16302, and XZ35305) obtained from Inner Mongolia Agricultural University of China were selected for the research, and *L. casei* 6117 and *Streptococcus thermophilus* GST-6 were also selected. The LAB strains stored at −80 °C were inoculated in MRS (de Man, Rogosa, and Sharpe) liquid medium [50] at 37 °C for 18 h for the recovery, and the strains after two generations of activation were used for follow-up experiments.

### 4.2. Lead Absorption Experiment

By dissolving appropriate quantities of lead nitrate in sterile ultrapure water and in MRS broth, 20-mg/L Pb^2+^ solutions were prepared. Ten LAB strains were collected by centrifugation at 8000× *g* for 20 min and washed twice with sterile ultrapure water; 0.6 g bacterial pellet was suspended in 50 mL lead solution, then cultured by shaking at 37 °C. At the corresponding time point, a 2-mL culture was centrifuged at 10,000× *g* for 10 min and the supernatant was used to detect the concentration of lead ions by ICP-MS (7700X, Agilent, Santa Clare, CA, USA). Each strain had three biological replicates. The heavy metal absorption rates were calculated according to the formulas described previously [51]. The metal concentrations in the solutions were determined at the beginning (C_0_) and end (C_e_) of the shaking period:

$$\mathrm{absorption}\;\mathrm{rates}\;(\%)\;=\;\frac{C_{0}-C_{e}}{C_{0}}\;\times \;100$$

### 4.3. Determination of Bacterial Tolerance to Lead 

The lead tolerance of each strain was determined by the minimum inhibitory concentration (MIC) approach [49]. After the lead nitrate solution was filtered with a 0.22-µm pore membrane, it was added to MRS solid medium. The lead ion concentrations were 100, 500, and 1000 mg/L. The MRS solid medium without lead was used as the control; 100 µL of cultured LAB strain was spotted in each plate. The LAB strains were cultured on the solid medium for 48 h at 37 °C. Each strain had three plates as biological replicates. The lowest concentration of lead that completely inhibited the growth was considered as the MIC.

### 4.4. Effect of Lead on the Growth of LAB

After the lead solution was filtered with a 0.22-µm pore membrane, it was added to MRS liquid medium. The lead ion concentration was 100 mg/L. The MRS liquid medium without lead was used as the control. The LAB strains were cultured by shaking in the liquid medium at 37 °C. Each strain had three biological replicates. At the corresponding time point, the growth of LAB was determined for absorbance of optical density 600nm (OD_600nm_) by spectrophotometer.

### 4.5. Preparation of Samples for Scanning Electron Microscopy (SEM) and Proteomic Analysis

LAB strains with excellent absorption/tolerance to lead were cultured in MRS liquid medium added lead ion at 100 mg/L, and the corresponding LAB strains cultured in normal MRS liquid medium were used as the control. The LAB strains after cultivating for 20 h were centrifuged at 7000× *g* for 15 min and washed twice with PBS buffer. The samples were used for the SEM (s4800, HITACHI, Takyo, Japan) observation and proteomic analysis. There were three biological replicates for each treatment.

### 4.6. SEM Observation

The samples were fixed with 2.5% glutaraldehyde and placed in 4 °C for the night. Then, the samples were dehydrated by gradient of 50%, 70%, and 100% ethanol. Finally, the samples were freeze-dried, sprayed with platinum. The surface morphological and elemental components of the strain were analyzed by scanning electron microscopy combined with energy dispersive X-ray spectrometer (SEM-EDS, Shimadzu, Manchester, UK).

### 4.7. Protein Extraction and Determination

Total protein was extracted from samples using a method as previously described [52]. The protein concentration was quantified by the bicinchoninc acid (BCA) method according to the instructions of the kit (BCA Protein Assay Kit, PC0020, Solarbio, Beijing, China). SDS-PAGE was used to analyze the protein samples and to evaluate the quality of the samples. The qualified protein samples were reduced by alkylation treatment. An equal amount of protein was taken from each sample for enzymatic hydrolysis, and the peptides were labeled with the iTRAQ reagent. The labeled peptides of six samples were mixed in equal amounts [53,54]. The mixed peptides were pre-separated by C18 reversed-phase chromatographic column and analyzed by Q Exactive mass spectrometer that was coupled with Easy-nLC 1200 (Thermo Fisher Scientific, San Jose, CA, USA). The Mascot 2.2 (Matrix Science, London, UK) and Proteome Discoverer 1.3 (Thermo Fisher Scientific, Waltha, MA, USA) software were used to simultaneously identify and quantify the raw data comparisons against *L. plantarum* WCFS1(Table S1).

The function of the identified proteins was annotated using Gene Ontology (GO) analysis, and the metabolomics pathway was analyzed using the Kyoto Encyclopedia of Genes and Genomes (KEGG) database.

### 4.8. Bioinformatic Analysis

MS/MS spectra were searched using ProteinDiscovererTM Software 2.1 against the Uniprot–*Lactobacillus plantarum* database and the decoy database with the following parameters. The highest score for a given peptide mass (best match to that predicted in the database) was used to identify parent proteins. For protein identification, a mass tolerance of 0.05 Da was permitted for intact peptide masses and of 0.1 Da was permitted for fragmented masses, with allowance for one missed cleavage upon trypsin digest. The parameters for protein searching were set as follows: tryptic digestion with up to two missed cleavages, carbamidomethylation of cysteines and the iTRAQ of *N*- terminus and lysine side chains of peptides as fixed modification, and oxidation of methionines and protein *N*-terminal acetylation as variable modifications. The quantitation protein ratios were weighted and normalized by the median ratio in Mascot.

### 4.9. Statistical Analysis

All the analyses were carried out in triplicate, and the values are represented as the mean values. Data were analyzed by analysis of variance (ANOVA) using SPSS 13.0 statistical package (SPSS Inc Chicago, Ill, USA). The results were considered to be statistically different at *p* < 0.05. Tukey test was used for comparing treatment means.

## Figures and Tables

**Table 1:** Lead tolerance of different lactic acid bacterial strains.

Stains	MIC (mg/L)
*L. plantarum* YW11	>1000
*L. plantarum* SKT109	>100
*L. plantarum* K25	>100
*L. plantarum* YNF-5	<100
*Lactococcus lactis* YNK-1-1	>100
*Lactococcus lactis* XZ16302	>100
*Lactococcus lactis* XZ35305	<100
*Lactococcus lactis* QH40-5	<100
*L. casei* 6117	>100
*S. thermophilus* GST-6	>100

MIC: minimum inhibitory concentration.

**Table 2:** Differentially expressed proteins between *L. plantarum* YW11 in Pb conditions and *L. plantarum* YW11 in Pb-free conditions.

Category	Accession	Description	Subcellular Localization	FC
Amino acid metabolism	A0A0R1VB59	dtd: d-aminoacyl-tRNA deacylase	cytoplasm	−1.57
Transporter	D7V9Y8	pstS: phosphate-binding protein	membrane	1.7
	A0A151G577	AYO51_05730: hemin ATP-binding cassette (ABC) transporter	—	1.54
Mismatch repair	A0A2S3U4Y4	xseB: exodeoxyribonuclease 7 small subunit	cytoplasm	1.52
Transcriptional regulation	A0A199QFG9	nrdR: transcriptional repressor	—	2.03
Membrane protein and cell surface protein	A0A199QM58	A0U96_08550: peptidoglycan-binding protein	—	2.17
	A0A2K7QYX4	A0U96_06145: peptidoglycan-binding protein	—	2.17
	A0A199QI49	A0U96_08550: adhesin	membrane	1.56
	A0A369UCN7	DVK84_07570: cell wall anchor domain-containing protein	—	1.76
	A0A162GCP0	Nizo2802_2963: membrane occupation and recognition nexus (MORN) motif family protein	membrane	1.64
Global stress response	A0A0L7Y2V1	A8704_12230: flavin mononucleotide (FMN)-binding protein	—	3.08
	A0A385PQP5	CFI98_11100: DNA replicationg protein D (DnaD) domain protein	—	1.84
	A0A1W6NPN7	BIZ32_04340: FMN-binding protein	membrane	1.57
	D7V968	adh: chaperonin 10 (GroES)-like protein	—	−1.78
	A0A0M0CIV7	hemH: ferrochelatase	cytoplasm	−1.51
	A0A0M0CJD8	AYO51_13390: macro domain ADP–ribose-binding module	—	−1.59
	A0A0R1UML2	FD10_GL000592: deoxycytidine (dCMP) deaminase	—	−2.22
	A0A1E3KN19	LPJSA22_03294: putative transposon Tn552 DNA-invertase bin3	—	−2.56
	A0A0R1V3I3	FD10_GL001348: anaerobic ribonucleoside-triphosphate reductase large subunit	—	−1.69
Extracellular protein	A0A0G9FAG5	AVR82_06000: extracellular protein	—	2.28
	A0A165XVF0	Nizo1839_1013: cell-shape-determining protein	—	1.55
	A0A2I0ZH16	CUR48_01040: Lysin motif (LysM) domain-containing protein	—	1.96
	A0A165P076	Nizo2802_0557: extracellular protein	—	1.58
Carbohydrate metabolism	A0A1S0RZ68	AVR82_00090: glycosyl hydrolase family	membrane	1.89
	T5JT98	N692_15475: formate acetyltransferase	cytoplasm	−1.82
Translation	A0A2S3U2L6	rplO: 50S ribosomal protein L15	—	−1.52
	A0A1A0DF73	rpmG: 50S ribosomal protein L33	intracellular	−1.56
	A0A199QKR6	rpsI: 50S ribosomal protein S9	—	−1.72
	T5K018	rplU: 50S ribosomal protein L21	intracellular	−1.75
	D7V8T5	rpmI: 50S ribosomal protein L35	intracellular	−1.82
	Q88XY4	rplW: 50S ribosomal protein L23	intracellular	−1.89
	U2WMY2	rpsU: 30S ribosomal protein S21	intracellular	−2.83
	A0A0G9FAQ2	ybaK: cys-tRNA(Pro)/Cys-tRNA(Cys) deacylase	—	−1.67
	A0A0R2GAU1	rsmG: ribosomal RNA small subunit methyltransferase G	cytoplasm	−1.49
Uncharacterized protein	A0A162GHW5	Lp19_2585	membrane	3.71
	A0A0G9F7Y4	DVK84_02520	—	2.29
	F9UU29	lp_0444	—	1.57
	A0A1S0RQZ5	AVR82_00885	membrane	1.74
	A0A165NJ25	Nizo2802_1443	—	1.56
	A0A0M4CUJ4	AVR82_13000	—	1.58
	M4KLN1	zj316_3034	—	1.56
	A0A386RBN5	CO218_15800	—	1.52
	A0A387DFR9	CFI62_00270	—	−1.64
	A0A0G9GNJ2	WP50_25770	membrane	−2.78

FC indicates fold change of each differentially expressed protein in the comparison of the treatment/control groups; — represents no prediction of subcellular localization.

## Supplementary Materials

Supplementary materials can be found at https://www.mdpi.com/1422-0067/20/22/5540/s1.
