# Supplementary material for: Evaluation and Proteomic Analysis of Lead Adsorption by Lactic Acid Bacteria
Source: Int J Mol Sci. 2019 Nov 6;20(22):5540. doi: 10.3390/ijms20225540 (PMC6888269; doi:10.3390/ijms20225540)
Supplement: Supplementary file 1 [file ijms-20-05540-s001.pdf]

International Journal of *Molecular Sciences*

MDPI

Supplementary

Evaluation and Proteomic Analysis of Lead Adsorption by Lactic Acid Bacteria

Shaoli Liu [1,2], Yi Zheng [1,2], Yimiao Ma [1,2], Abid Sarwar [1,2], Xiao Zhao [1,2], Tianqi Luo [1,2] and Zhennai Yang [1,2,*]

[1] Beijing Advanced Innovation Center for Food Nutrition and Human Health, Beijing Technology and Business University, Beijing 100048, China;

[2] Beijing Engineering and Technology Research Center of Food Additives, Beijing Technology and Business University, Beijing 100048, China

* Correspondence: yangzhennai@th.btbu.edu.cn

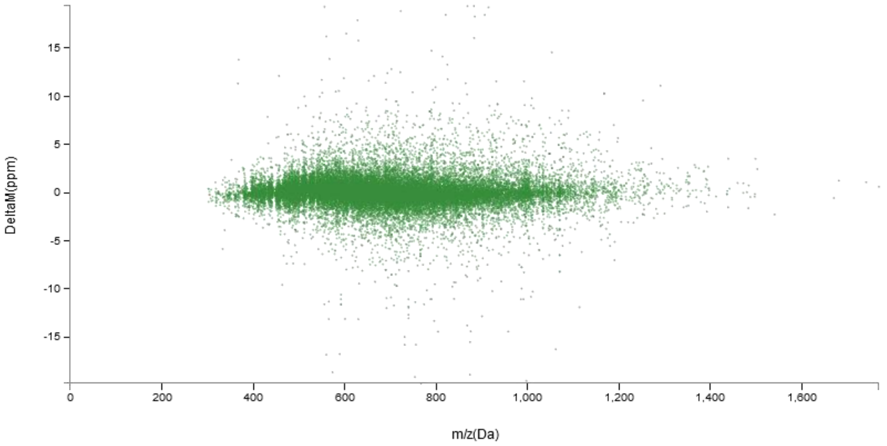

Figure 1. The distribution of peptide matching error. The figure shows the error distribution between the true value and the theoretical value of the relative molecular weight of all matched peptides.

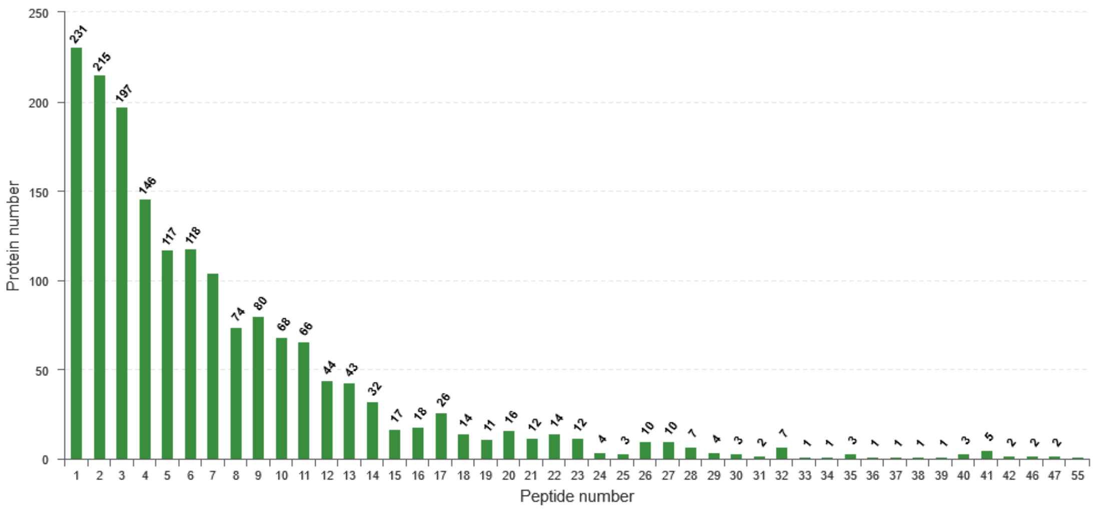

Figure 2. Peptide number distribution. The figure shows the distribution of the number of peptides in the identified protein. The abscissa representing the number of peptides covering the protein and the ordinate representing the number of proteins.

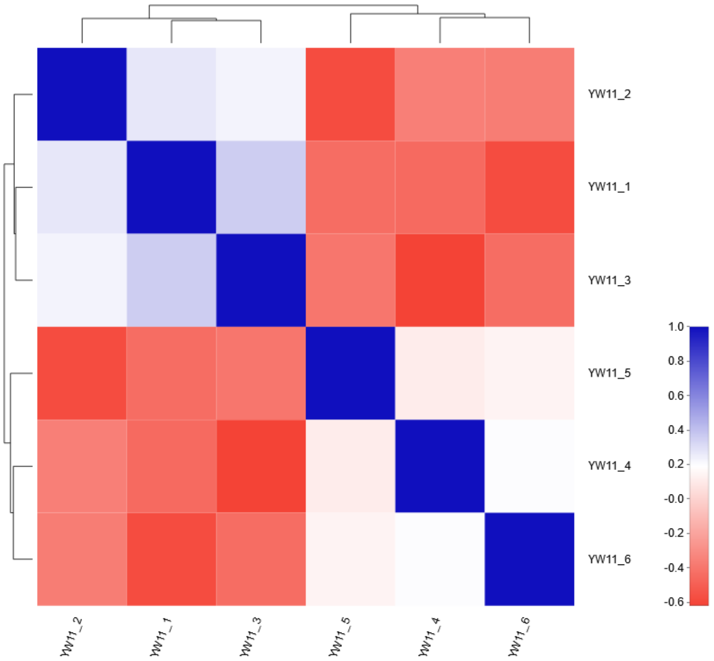

Figure 3. Correlation between samples of YW11. YW11-1,2,3: YW11 in lead - free medium; YW11-4,5,6: YW11 in medium containing lead ions (100mg/L).

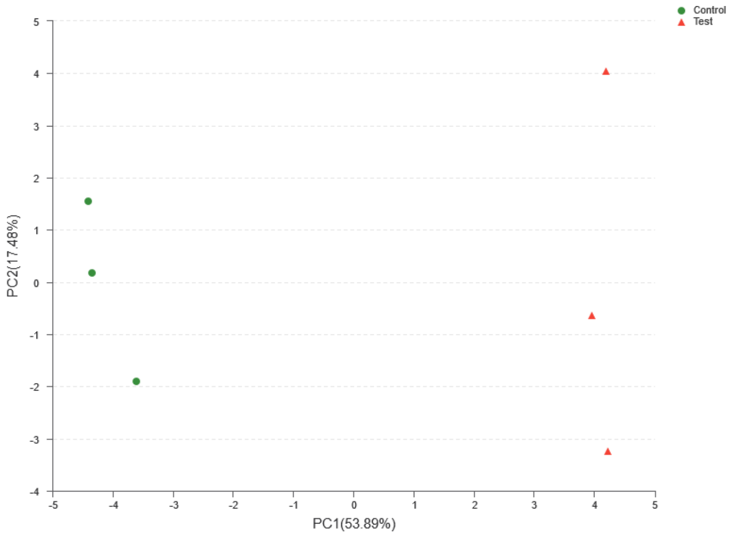

Figure 4. PCA analysis of YW11 proteins. Control: YW11 in lead - free medium; Test: YW11 in medium containing lead ions (100mg/L).

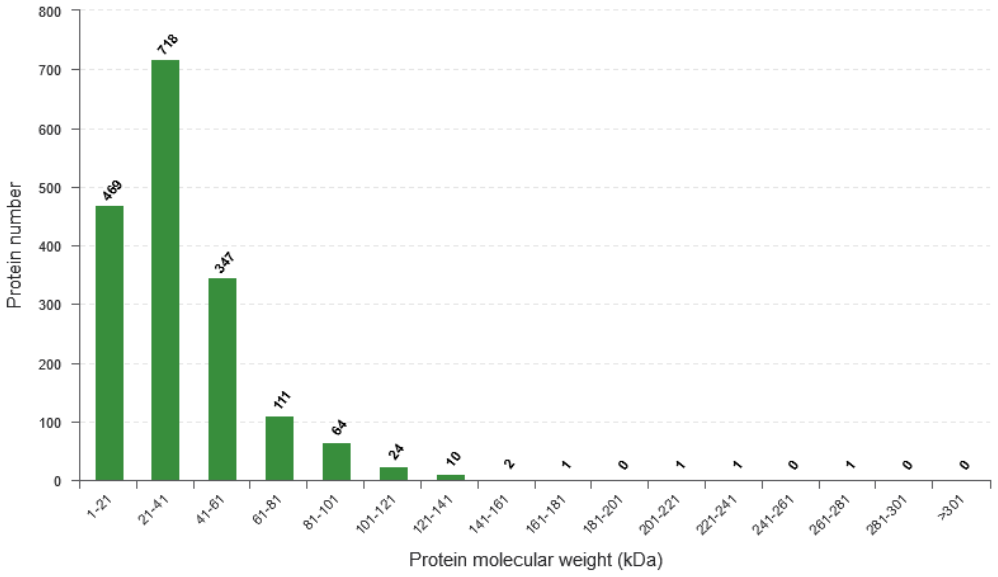

Figure 5. Peptide molecular weight distribution. The figure shows the molecular weight distribution of identified proteins. The abscissa is the distribution range of molecular weight of proteins, and the ordinate is the number of proteins with corresponding molecular weight.

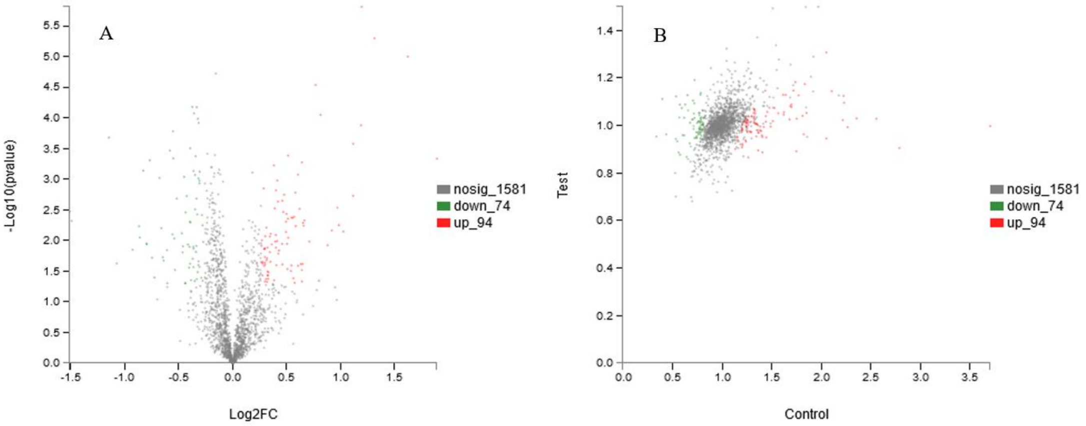

Figure 6. Significant analysis of differentially expressed proteins. (**A**)Volcano analysis of samples treated with lead ions vs the control.; (**B**) Scatter analysis of samples treated with lead ions vs the control.

Table S1. Information of identified protein

Accession	Control	Test	pvalue	fc	regulate	significant	description
A0A0R1VB59	0.959	0.6157	0.02125	0.642	down	yes	D-aminoacyl-tRNA deacylase OS=Lactobacillus plantarum subsp. argentoratensis DSM 16365 OX=1423831 GN=dtd PE=3 SV=1
D7V9Y8	1.029	1.753	2.90E-05	1.704	up	yes	Phosphate binding protein OS=Lactobacillus plantarum subsp. plantarum ATCC 14917 = JCM 1149 = CGMCC 1.2437 OX=525338 GN=pstS PE=4 SV=1
A0A151G577	1.056	1.623	0.003042	1.537	up	yes	Hemin ABC transporter ATP-binding protein OS=Lactobacillus plantarum OX=1590 GN=AYO51_05730 PE=4 SV=1
A0A2S3U4Y4	0.95	1.445	0.0008392	1.521	up	yes	Exodeoxyribonuclease 7 small subunit OS=Lactobacillus plantarum subsp. plantarum OX=337330 GN=xseB PE=3 SV=1
A0A199QFG9	1.096	2.23	0.007091	2.035	up	yes	Transcriptional repressor NrdR OS=Lactobacillus plantarum OX=1590 GN=nrdR PE=3 SV=1
A0A199QM58	1.023	2.216	0.001862	2.166	up	yes	Peptidoglycan-binding protein OS=Lactobacillus plantarum OX=1590 GN=A0U96_08550 PE=4 SV=1
A0A2K7QYX4	0.946	2.051	0.0002637	2.168	up	yes	Peptidoglycan-binding protein OS=Lactobacillus plantarum OX=1590 GN=A8704_14660 PE=4 SV=1
A0A199QI49	1.175	1.834	0.04642	1.561	up	yes	Adhesin OS=Lactobacillus plantarum OX=1590 GN=A0U96_06145 PE=4 SV=1
A0A369UCN7	1.053	1.851	8.92E-05	1.758	up	yes	LPXTG cell wall anchor domain-containing protein OS=Lactobacillus plantarum OX=1590 GN=DVK84_07570 PE=4 SV=1
A0A162GCP0	1.026	1.679	0.01033	1.636	up	yes	MORN motif family protein OS=Lactobacillus plantarum OX=1590 GN=Nizo2802_2963 PE=4 SV=1
A0A0L7Y2V1	0.9057	2.788	9.98E-06	3.078	up	yes	FMN-binding protein OS=Lactobacillus plantarum OX=1590 GN=A8704_12230 PE=4 SV=1
A0A385PQP5	1.145	2.105	0.01188	1.838	up	yes	DnaD domain protein OS=Lactobacillus plantarum OX=1590 GN=CFI98_11100 PE=4 SV=1
A0A1W6NPN7	1.127	1.771	0.005153	1.571	up	yes	FMN-binding protein OS=Lactobacillus plantarum OX=1590 GN=BIZ32_04340 PE=4 SV=1
D7V968	0.9437	0.5317	0.0007232	0.563	down	yes	GroES-like protein OS=Lactobacillus plantarum subsp. plantarum ATCC 14917 = JCM 1149 = CGMCC 1.2437 OX=525338 GN=adh PE=3 SV=1
A0A0M0CIV7	0.877	0.5753	0.01433	0.656	down	yes	Ferrochelatase OS=Lactobacillus plantarum OX=1590 GN=hemH PE=3 SV=1
A0A0M0CJD8	0.8867	0.561	0.006253	0.633	down	yes	Macro domain ADP-ribose binding module OS=Lactobacillus plantarum OX=1590 GN=AYO51_13390 PE=4 SV=1
A0A0R1UML2	0.9633	0.4353	0.0002086	0.452	down	yes	dCMP deaminase OS=Lactobacillus plantarum subsp. argentoratensis DSM 16365 OX=1423831 GN=FD10_GL000592 PE=4 SV=1
A0A1E3KN19	0.954	0.3353	0.003346	0.351	down	yes	Putative transposon Tn552 DNA-invertase bin3 OS=Lactobacillus plantarum OX=1590 GN=LPJSA22_03294 PE=4 SV=1
A0A0R1V3I3	0.9337	0.5483	0.0004908	0.587	down	yes	Anaerobic ribonucleoside-triphosphate reductase large subunit OS=Lactobacillus plantarum subsp. argentoratensis DSM 16365 OX=1423831 GN=FD10_GL001348 PE=4 SV=1
A0A0G9FAG5	0.993	2.265	0.0001318	2.281	up	yes	Extracellular protein OS=Lactobacillus plantarum OX=1590 GN=AVR82_06000 PE=4 SV=1
A0A165XVF0	1.046	1.62	0.02415	1.549	up	yes	Cell shape-determining protein MreC OS=Lactobacillus plantarum OX=1590 GN=Nizo1839_1013 PE=3 SV=1
A0A2I0ZH16	0.8923	1.748	0.002902	1.959	up	yes	LysM domain-containing protein OS=Lactobacillus plantarum OX=1590 GN=CUR48_01040 PE=4 SV=1
A0A165P076	0.8873	1.406	0.004675	1.585	up	yes	Extracellular protein OS=Lactobacillus plantarum OX=1590 GN=Nizo2802_0557 PE=4 SV=1
A0A1S0RZ68	0.9633	1.822	0.00683	1.891	up	yes	Glycosyl hydrolase family 8 OS=Lactobacillus plantarum OX=1590 GN=AVR82_00090 PE=4 SV=1
T5JT98	1.034	0.567	0.005848	0.548	down	yes	Formate acetyltransferase OS=Lactobacillus plantarum EGD-AQ4 OX=1382301 GN=N692_15475 PE=4 SV=1
A0A2S3U2L6	1.094	0.7187	0.04949	0.657	down	yes	50S ribosomal protein L15 OS=Lactobacillus plantarum subsp. plantarum OX=337330 GN=rplO PE=3 SV=1
A0A1A0DF73	1.105	0.7057	0.01869	0.639	down	yes	50S ribosomal protein L33 OS=Lactobacillus plantarum OX=1590 GN=rpmG PE=3 SV=1
A0A199QKR6	1.128	0.65	0.01155	0.576	down	yes	30S ribosomal protein S9 OS=Lactobacillus plantarum OX=1590 GN=rpsI PE=3 SV=1
T5K018	1.089	0.626	0.01114	0.575	down	yes	50S ribosomal protein L21 OS=Lactobacillus plantarum EGD-AQ4 OX=1382301 GN=rplU PE=3 SV=1
D7V8T5	1.074	0.5917	0.008909	0.551	down	yes	50S ribosomal protein L35 OS=Lactobacillus plantarum subsp. plantarum ATCC 14917 = JCM 1149 = CGMCC 1.2437 OX=525338 GN=rpmI PE=3 SV=1
Q88XY4	1.088	0.572	0.01408	0.526	down	yes	50S ribosomal protein L23 OS=Lactobacillus plantarum (strain ATCC BAA-793 / NCIMB 8826 / WCFS1) OX=220668 GN=rplW PE=3 SV=1
U2WMY2	1.123	0.5337	0.02365	0.475	down	yes	30S ribosomal protein S21 OS=Lactobacillus plantarum AY01 OX=1358413 GN=rpsU PE=3 SV=1
A0A0G9FAQ2	1.033	0.6157	0.01903	0.596	down	yes	Cys-tRNA(Pro)/Cys-tRNA(Cys) deacylase OS=Lactobacillus plantarum OX=1590 GN=ybaK PE=3 SV=1
A0A0R2GAU1	1.012	0.6763	0.0003416	0.668	down	yes	Ribosomal RNA small subunit methyltransferase G OS=Lactobacillus plantarum OX=1590 GN=rsmG PE=3 SV=1
A0A162GHW5	0.9977	3.705	0.0004604	3.714	up	yes	Uncharacterized protein OS=Lactobacillus plantarum OX=1590 GN=Lp19_2585 PE=4 SV=1
A0A0G9F7Y4	1.03	2.357	1.54E-06	2.288	up	yes	Uncharacterized protein OS=Lactobacillus plantarum OX=1590 GN=DVK84_02520 PE=4 SV=1
F9UU29	1.308	2.05	0.0241	1.567	up	yes	Uncharacterized protein OS=Lactobacillus plantarum (strain ATCC BAA-793 / NCIMB 8826 / WCFS1) OX=220668 GN=lp_0444 PE=4 SV=1
A0A1S0RQZ5	1.041	1.811	0.04515	1.740	up	yes	Uncharacterized protein OS=Lactobacillus plantarum OX=1590 GN=AVR82_00885 PE=4 SV=1
A0A165NJ25	1.089	1.699	0.0005295	1.560	up	yes	Uncharacterized protein OS=Lactobacillus plantarum OX=1590 GN=Nizo2802_1443 PE=3 SV=1
A0A0M4CUJ4	1.078	1.7	0.005721	1.577	up	yes	Uncharacterized protein OS=Lactobacillus plantarum OX=1590 GN=AVR82_13000 PE=4 SV=1
M4KLN1	1.082	1.687	0.03018	1.559	up	yes	Uncharacterized protein OS=Lactobacillus plantarum ZJ316 OX=1284663 GN=zj316_3034 PE=4 SV=1
A0A386RBN5	0.8957	1.363	0.02574	1.522	up	yes	Uncharacterized protein OS=Lactobacillus plantarum OX=1590 GN=CO218_15800 PE=4 SV=1
A0A387DFR9	1.192	0.7213	0.04001	0.605	down	yes	Uncharacterized protein OS=Lactobacillus plantarum OX=1590 GN=CFI62_00270 PE=4 SV=1
A0A0G9GNJ2	1.112	0.395	0.004783	0.355	down	yes	Uncharacterized protein OS=Lactobacillus plantarum OX=1590 GN=WP50_25770 PE=4 SV=1
